# Thoracolumbar injuries: operative treatment: indications, techniques, timing and implant removal. Current practice

**DOI:** 10.1007/s00068-024-02602-y

**Published:** 2024-08-27

**Authors:** Frank Bloemers, Marko Jug, Christoph Nau, Radko Komadina, Hans Christoph Pape, Klaus Wendt

**Affiliations:** 1 Amsterdam University Medical Centres, Vrije Universiteit Amsterdam, Amsterdam, Netherlands; 2 University of Ljubljana, Ljubljana, Slovenia; 3 University Hospital Frankfurt, Goethe University, Frankfurt, Germany; 4 University Hospital of Zürich, University of Zürich, Zürich, Switzerland; 5 University of Groningen, Groningen, Netherlands

**Keywords:** Spinal fractures, Minimal invasive, Timing of surgery, Implant removal, Minimal invasive, Techniques, Wervelfrakturen, Chirurgische techniek, Minimaal invasief, Timing van operatie, Verwijderen materiaal, Wirbelfrakturen, Operationstechnik, Minimalinvasiv, Timing of surgery, Implant removal

## Abstract

The operative treatment of thoracolumbar fractures is a rapidly evolving improvement in the care of patients with this injury after trauma. This article describes the different techniques and principles. Considerations and methods of treatment are scientifically addressed and illustrated according to the classification and severity of the fracture pattern. The use of computer navigation and optimisation of minimally invasive techniques is inevitable. The timing of surgery as well the removal of the material after fracture healing are also discussed. The operative treatment of spinal fractures is emerging and there is still much more knowledge to gain.

## Introduction

Thoracolumbar spine injuries can have severe implications on an individual’s mobility and quality of life. These injuries are often the result of trauma, such as falls, motor vehicle accidents, or sports-related incidents. While conservative management can be effective in many cases, surgical treatment has emerged as an important approach to complex thoracolumbar spine injuries. The purpose of this article is to explore the advancements and considerations associated with operative interventions for these injuries. As Hippocrates noted over 2 millennia ago, conservative fracture treatment has long been the standard treatment of care. He was the first to describe the spine as kind of chain stabilised by anterior and posterior ligaments. He stated that spinal fractures without neurological deficit recover quickly with conservative treatment [1]. This basic principle is still valid, but complications such as malunion, non-union and especially post-traumatic kyphosis can lead to sagittal imbalance and significant morbidity. For a long time bracing was the only treatment available. The first successful surgical fracture stabilisation of the spine was performed in 1911. The posterior column was fixed by wrapping wires around it [2]. The tuberculosis pandemic gave a further boost to spinal surgery. The introduction of rods in the 1950s formed the basis of scoliosis surgery and it was not until the late 20th century that the first trauma cases were operated on. Since then, there has been a rapid development of implants and surgical techniques to stabilise the injured spine [3]. The aim of surgical treatment is to achieve anatomical reconstruction of the spine with immediate stability and relief of any spinal cord compression [4].

## Technique and principles

The goal of stabilisation can be instrumentation or fusion (spondylodesis). Instrumentation is defined as posterior or anterior stabilisation without the definitive fusion of the facet joints. Fusion or “spondylodesis” is defined as the permanent fusion of a mobile segment. This can be achieved either through an anterior or a posterior approach or by a combination of both. The technique of posterior fusion includes decortication of the interbody joint, placement of autogenous or allogenic bone graft or use of osteoconductive and/or osteoinductive bone substitutes. Anterior reconstruction is defined as the anatomical restoration of the ventral column with the use of implants (cages, ventral instrumentation), grafts, or other materials. This can also be performed through a posterior approach.

The selection ofthe most appropiate technique is contingent upon the fracture classification, the presence of neurological deficits and the specific anatomical considerations of the patient. In a patient with diffuse idiopathic skeletal hyperostosis (DISH) spine, the mechanical aspects are distinct from those ofa relative young spine with greater movability. The operative treatment should be tailored to the classification of the vertebral fracture and the severity of the concomitant injuries of the patient [9, 30]. The right classification mandates the best treatment.

The classification of the fractures in the thoracolumbar region has only recently been developed into a comprehensive classification system [3]. The AO Spine Thoracolumbar Injury Classification treatment algorithm is a valid and useful tool in the context of daily practice. Internationally, this comprehensive classification is incorporated into national treatment guidelines. In general, a score of greater than 5 points indicates the necessity for surgical stabilisation [3, 9].

The surgical management of spinal fractures encompasses a variety of surgical techniques.

Dorsal (internal fixator, pedicle screws).

- open versus minimal (less) invasive.
- short versus long trajectory.
- spondylodesis.
- cement augmentation.

Ventral.

- open versus minimal (less) invasive.
- internal fixator.
- cage with or without internal fixator.
- 360° (combination dorsal/ventral).

Hinojosa-Gonzalez et al. conducted a meta-analysis to compare the different approaches. The findings suggest that the posterior approach technique may be a more optimal option for surgically managing thoracolumbar fractures. A number of advantages were identified, including a shorter operative time, decreased blood loss, reduced Length of Stay (LoS), and a lower incidence of complication. Further high-quality evidence is required to inform the selection of the optimal surgical approach, taking into account several factors, such as surgeon experience and resource availability [31].

Different solutions can be emplyed to enhance stability. For example, the stability of the spinal segment can be enhanced by stabilizing either a greater or lesser extent. It is recommended that long segmental instrumentation should be used at the upper and middle thoracic spine (above Th 10). At the thoracolumbar junction and the lumbar spine short segmental stabilisation is typically sufficient with better clinical outcomes [8–11, 13].

In a prospective study Zühtü Özbek et al. concluded that the number of patients with fusion was significantly greater in the short segment including fractured level (SSIFL) group compared with the long segment (LS) group. There was a significant reduction of the clinical scores of patients who had fusion compared with the fusion-free group.However, no radiologically significant differences were identified. Moreover, no significant difference were observed between the SSIFL and LS groups in terms of the 2-year radiologic and clinical follow-up results [12].

The utilisation of intermediate screws at the fracture level has been demostrated to enhance construct stability and to minimise reduction loss [5, 14].

Monoaxial implants should be used if no additional anterior stabilization is performed. In contrast, loss of reduction is more likely in patients instrumented with polyaxial screws. Transverse connecting rods can be used to increase the stability [8].

Patients will benefit from a minimally invasive anterior approach by reducing morbidity and improves mobility [6,7,^13^].

The use of cement augmentation with PMMA (polymethyl methacrylate) cement is a valuable technique in patients with reduced bone quality. It is not recommended for use in young patients with a healthy bone stock [8].

The operative management of thoracolumbar burst fractures without neurologic deficit may improve residual kyphosis, but does not appear to improve pain or function at an average of four years after injury. Furthermore, itis associated with higher complication rates and costs [15].

In a prospective, randomized study of thoracolumbar burst fractures Wood et al. found that operative treatment did not achieve superior outcomes compared to non-operative treatment in neurologically intact patients [18]. In contrast, Siebenga et al. reported that that thoracolumbar burst (AO Type A3) fractures without neurologic deficit managed by short-segment posterior stabilization had better radiographic outcomes than nonsurgical treated fractures. However, the functional outcome was the same [19].

The precise positioning of pedicle screws is of paramount importancein the surgical the procedure. Malpositioning can result in a reduction in constructinstability or even in iatrogenic spinal cord injuries.

The introduction of computer assisted navigation systems has enhance the accuracy of pedicle screw placement while simultaneously reducing the radiation exposure to the patient and the operating room personnel during the procedure. Postoperative infections are relatively uncommon compared to other trauma orthopedic procedures. Minimally invasive surgery (MIS) has gained prominence due to the reduction of postoperative morbidity. In a retrospective, single-center study Hayoun et al. concluded that percutaneous surgery for thoracolumbar fractures results in less blood loss than conventional open surgery. The clinical and radiological results are comparable to those of conventional open surgery. The mean length of hospital stay is shorter and the accuracy of pedicle screw placement is higher with percutaneous surgery [30, 32]. Russo et al. concluded that both the open and minimally invasive approaches provide safe and effective treatment for thoracolumbar burst fractures However, there is currently no clear evidence that a percutaneous approach is superior in long-term patient-reported outcomes or radiographic parameters. Although percutaneous approaches demonstrate decreased blood loss and operative time, an open approach remains a safe and effective treatment option for thoracolumbar burst fractures without neurological injury [32].

Further techniques such as video-assisted thoracoscopic surgery (VATS) facilitate anterior stabilization with reduced postoperative complications [6–8]. This approach is beneficial for achieving 360 fixation in complex cases. But also in cases with nonunion and specific fractures like AO A2 this approach is beneficial. The utilisation of MIS for anterior stabilization encompasses the implementation of cages, vertebral stenting and anterior plate fixation. Also anterior stabilization and prevention of vertebral body collapse can be prevented by vertebral cementing, which is often combined with balloon technique or vertebral expandable stenting. Favorable long term outcome are reported worldwide [6]. When appropiate, the aforementioned procedures can be staged and combined with anterior procedures following posterior interventions.

The incidence of complications associated with operative treatment is relatively low, concerning wound infections. Moreover, antibiotic treatment is due to the good vascularization of the bone and surrounding soft tissue, mostly successful.

### Timing of operative treatment

It is necessary to distinguishbetween a spinal fracture with or without a neurological deficit.

## Thoracolumbar fractures without spinal cord injury

A number of systemic reviews have been conducted on the timing of surgery in the context of thoracolumbar fractures. Bellabarba et al. concludes that ideally, patients with unstable thoracic fractures should undergo early (72 h) stabilization of their injury to reduce morbidity and, possibly, even mortality [18, 20]. In a review of 10 studies with 2512 patients Dan Xing et al. reached the same conclusion. The early stabilization shortened the hospital length of stay, intensive care unit length of stay, ventilator days and reduced morbidity and hospital expenses particularly for patients with thoracic fractures. Nevertheless, reduced morbidity and hospital expenses were not replicated with stabilisation of lumbar fractures [21].

Boakye et al. classified in a retrospective study patients as having early (< 72 h) or late (> 72 h) surgery. Early surgery (< 72 h) for traumatic thoracic/thoracolumbar fractures was associated with a significantly lower overall complication rate (including cardiac, thromboembolic, and respiratory complications), and a shorter hospital stay. The in-hospital charges were found to be significantly lower ($38,120 difference) in the early surgery group. Multivariate analysis identified time to surgery as the most significant predictor of in-hospital complications. However age, medical comorbidities, and injury severity score were also independently associated with increased complications. We reinforce the beneficial impact of early spinal surgery (prior to 72 h), preferable within 48 hours [22].

## Thoracolumbar fractures with spinal cord injury

With regard to the timing of operative management, high-quality studies comparing early and delayed intervention are lacking. Based on the evidence regarding cervical spine injury it can be postulated that early intervention would also be beneficial for neurological recovery in these patients [21, 23]. In the event of a spinal cord injury in a patient receiving care in a emergency room, the trauma team leader should immediately contact the on call spinal surgeon on call at the trauma unit or nearest major trauma centre. [24]. Patients presenting with neurologic deficits caused by traumatic spinal canal stenosis should be treated as an emergency [17].

Fehlings et al. stated that there are currently no standards regarding the role and timing of decompression in acute SCI. They recommended urgent decompression of bilateral locked facets be performed in patients with incomplete tetraplegia neurologic deterioration. Urgent decompression in acute cervical spinal cord injury remains a reasonable practice option and can be performed safely. Emerging evidence suggests that surgical intervention within 24 h may reduce length of intensive care [24]. Surgical decompression within 24 h of acute spinal cord injury has been associated with improved sensorimotor recovery. The initial 24–36 h following an acute spinal cord injury appears to represent a crucial time window during which decompressive surgery may facilitate optimal neurological recovery. [24].

Early surgery and severity of initial injury (complete [ASIA A] vs. incomplete spinal cord injury [ASIA B-D]) were found to significantly influence the potential for neurologic improvement (P ¼ 0.004 and *P* <.0001, respectively) [20].

In conclusion thoracolumbar fractures with spinal cord injury should be treated as an emergency. There is mounting evidence that an operation within 24 h of injury results in neurological improvement.

## Removal of material

In general, no hard indications are existing for standard removal of material. Dorsal stabilisation by the use of screws and rods can result in stiffness and impaired movement of the spine. Also, when pedicle screws pronounce dorsally some patients complaints of muscle reactions and irritation of the skin. In young patients, approximately 10 months after injury the fractured vertebral and ligaments have healed sufficiently for the removal of material to be performed safely [26]. It is evident that material failure, predominantly screw breakage, can also serve as a rationale for removal when the patient is symptomatic.

One year after implantation, degenerative changes in the discus typically result in a reduction of the intervertebral disc space, arthrosis of the intervertebral joints and a reduced movement of the fixated segment.In literature, it has been reported implant breakage occurs in approximately onethird of the cases where the implant was not routinely removed after eight years [25].

Smits et al. conducted a retrospective analysis of 102 patients who had undergone removal of posterior osteosynthetic material following a fracture in the Th-L spine after 2 to 15 years, with a median of 7 years after the implantation. They found a slightly increased angle of the kyphosis, which was not clinically significant. In the majority of cases, the subjects reported a subjective improvement following the operative removal of the material. Conversely, only 11% of subjects reported a subjective exacerbation after the removal of the implant [26].

On average, five years following the implant, at least one screw was discovered to have broken in 36% of the patients from the group that was not advised to have a routine removal of the implant, while in the group with the routine implant removal, there were no cases of broken screws. There were no significant differences between the groups regarding collapse of damaged vertebrae, reduced intervertebral discs or increased kyphotic angles. No notable alterations were observed in the radiological and functional outcomes. [27].

In their meta-analysis, Kweh et al. did not find any statistically significant objective differences between cohorts of patients who had their implant electively removed after the fracture had healed and the cohorts where the implant was retained. A subjective improvement in sensation was observed in younger patients following implant removal, although this was not accompanied by a statistically significant reduction in the primarily corrected kyphotic angle [28].

Jug et al. expect there is a bigger benefit in removing the implant in younger patients and with longer fixations that take into account several moving segments at least 12 months after the initial procedure, and after the fracture has radiologically healed [29]. In cases of ankylosing spondylitis and in older patients, it is not recommended to remove the implant without a justified clinical indication [30].

Although the majority of patients with symptomatic and asymptomatic implants report a subjective improvement following removal, and despite the low incidence of postoperative complications the literature recommends that each patient be treated individually, with a thorough examination. Even discovering the event of the discovery of an individual broken screw, it is not necessary to proceed with its removal. Objective indications for the removal of the implant are infection, migration or endangering neurological structures, as well as issues related to the spine’s flexibility with long fixations. In the end, however, the patient’s decision must be respected [29].

## To illustrate the workup and surgical techniques for stabilization, four cases are described

### Case 1

A 25 year old male sustained two TL AO A3 fractures following a motor vehicle accident The patient’s hands also sustained complex fractures, necessitating multiple surgical procedures. The lung concussion was managed with a conservative approach. The spine was stabilized by percutaneous dorsal instrumentation in T10 and L1. In order to provide additional stabilisation, the fractured vertebrae were utilised. Given the comminuted nature of the fractures, it was deemed inadvisable to perform kyphoplasty with cementation anteriorly. The suboptimal correction of the kyphosis was accepted. Prior to the removal of the material in 10 months post-operatively, a CT scan was performed to confirm the healing of the fractures.


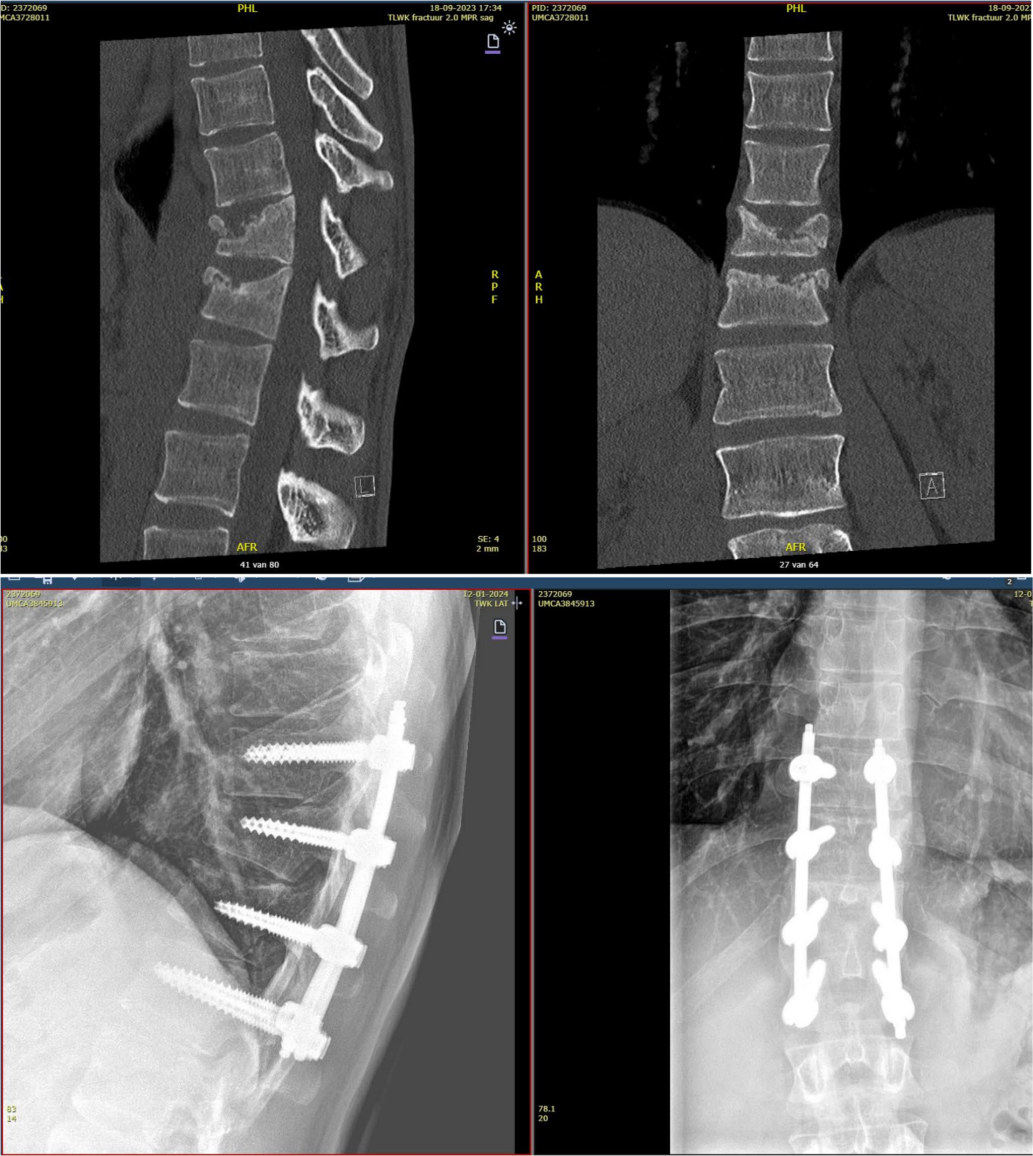


### Case 2

A 70 year old woman was presented in the trauma room following a high fall from the stairs. After assessment according to the ATLS, a TL AO A4 fracture was diagnosed. Fortunately no neurological symptoms were present. A dorsal percutaneous stabilization and transpedicular stenting with cementing procedure was performed on the fractured T12. During the cementing some leakage in the pedicels were seen, but not in the spinal canal. Due to the cemented T11 a short segment stabilization was possible. The A1 fractured L2 was treated conservatively.


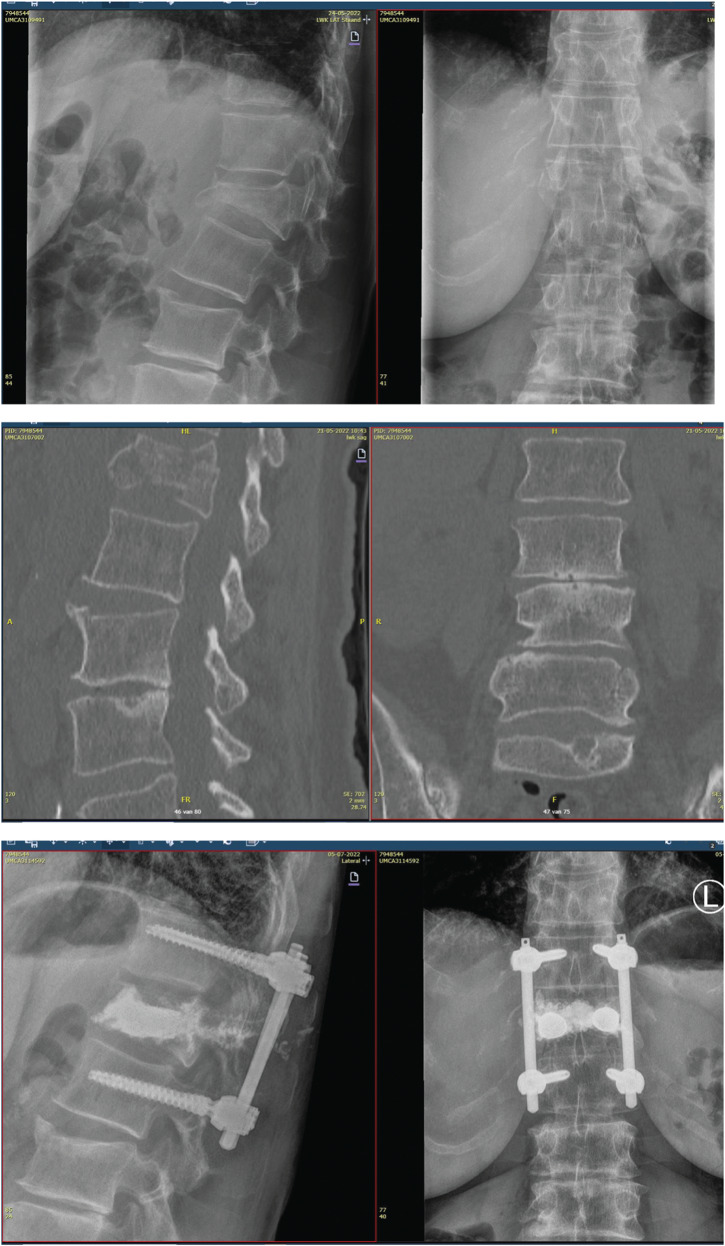


### Case 3

A 60 years-old man sustained a fall from aladder while engaged in trimming a treein the garden. Following a ct-scan, a B1 type fracture of T11 was diagnosed. No neurological deficit was identified. Given that a portion of the spine exhibited DISH and that more adjacent vertebrae were A3 fractured, two level up en two down were used to dorsally fixate the spinal column.


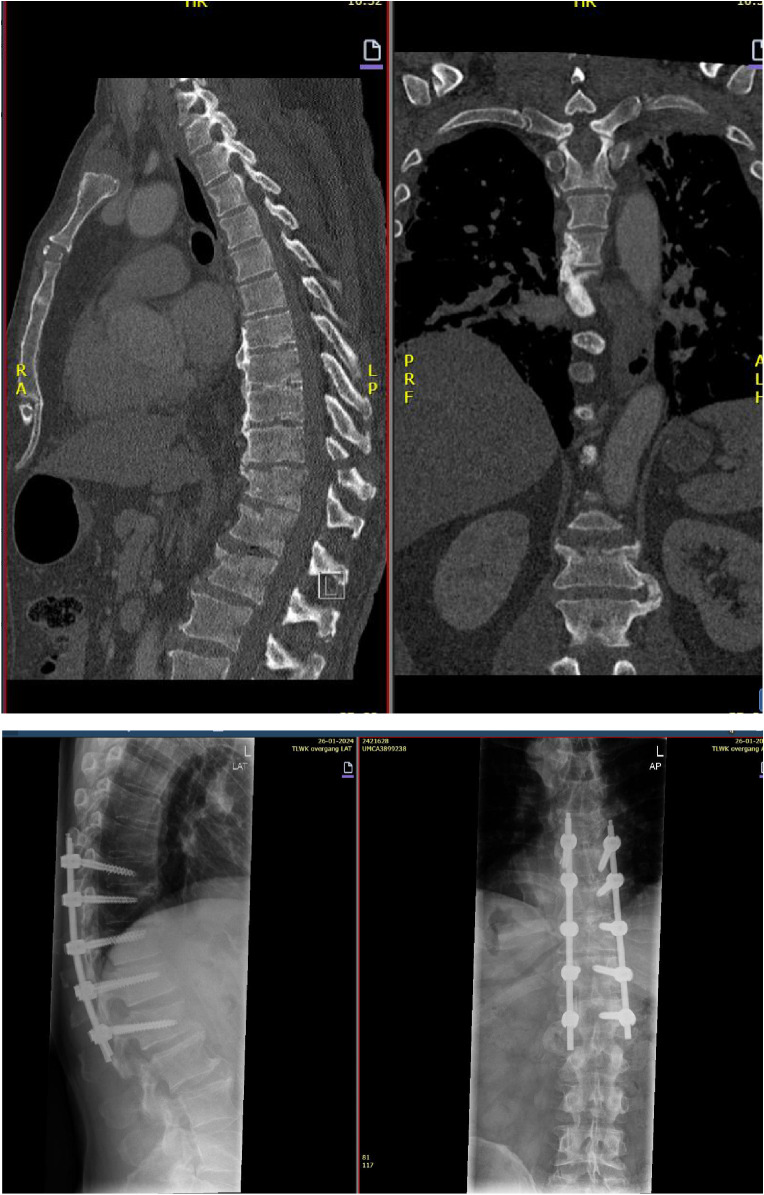


### Case 4

A 32 year old male, Motor Vehicle Accident, was presented with a paraplegia resulting from a C/A4 type T11 fracture. Following a CT scan an MRI was made to assess the extend of spinal damage and ligamentous involvement. An open reduction and dorsal stabilization was performed, followed by a laminectomy at T11 level. Given the multiple injuries sustained by this polytrauma, closely monitor of the fracture healing is mandatory. In the event of non union, 360 stabilisation, for instance with the use of an anterior cage is advised.


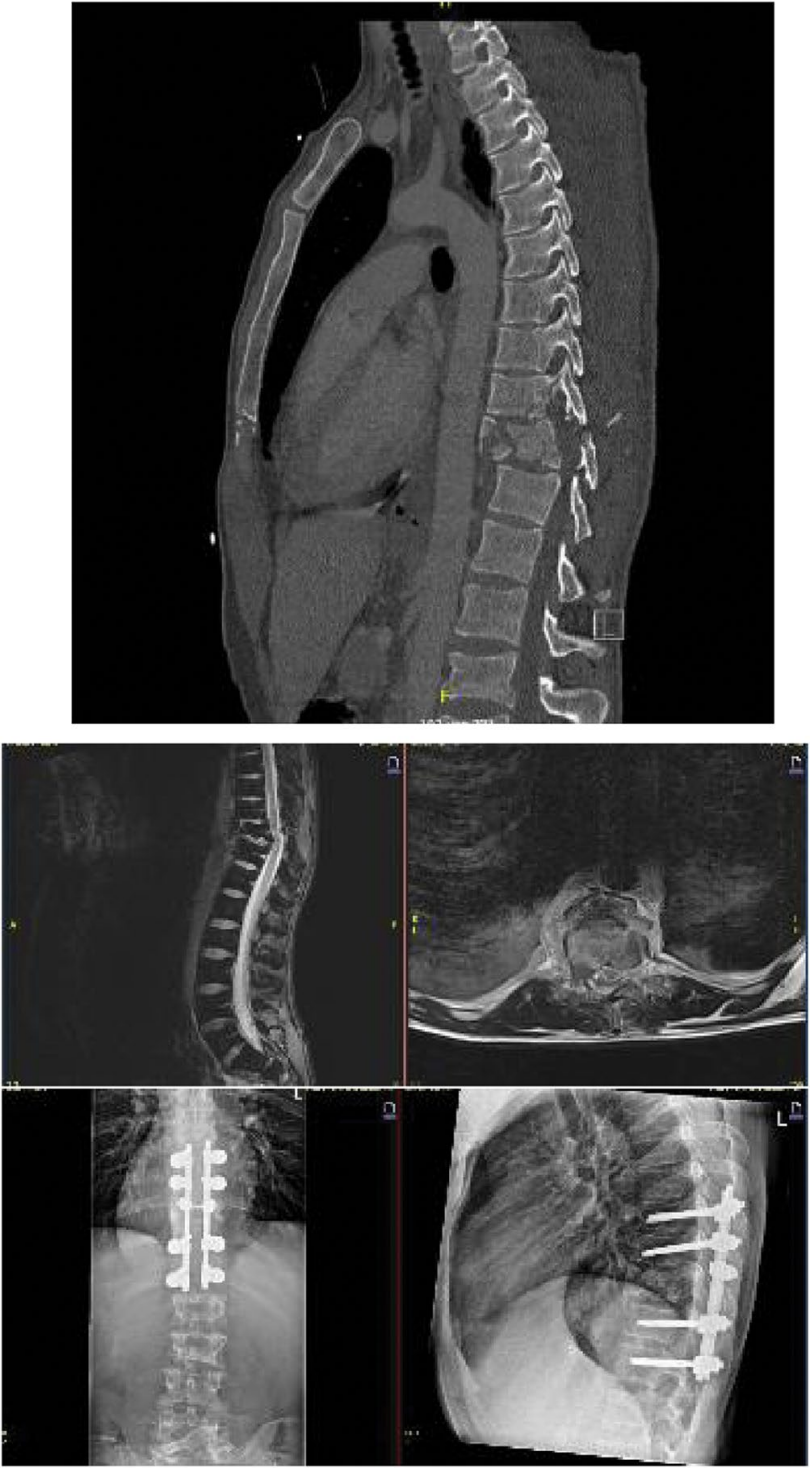


## Conclusion

In the aforementioned and illustrated trauma cases, different mechanism of injury are described. No surgical treatment in spine is strongly evidence based yet, however the scientific evidence is growing to support the use of the different techniques.

The operative treatment of spinal fractures is a dynamic field characterized by technological advancements and a commitment to patient centered care. The selection of surgical technique is contingent upon a comprehensive assessment of the fracture, neurological status and thepatients comorbidities. It is evident that a shared decision making approach is imperative. As the field of spinal surgery progresses, it is anticipated that emerging technologies such as computer assisted navigation systems will further enhance the precision and outcomes these procedures. A growing number of increasing cohort studies on advanced, minimally invasive procedures, are reporting good results and favorable patient- related outcomes.

## Data Availability

No datasets were generated or analysed during the current study.
